# Supplementary material for: Ribozyme-catalysed RNA synthesis using triplet building blocks
Source: eLife. 2018 May 15;7:e35255. doi: 10.7554/eLife.35255 (PMC6003772; doi:10.7554/eLife.35255)
Supplement: Supplementary file 1. — All sequences are written in a 5’-to-3’ direction, and generated by GMP transcription of the corresponding PCR-generated dsDNA of the sequence downstream of 5T7 sequence duplex. All transcripts were PAGE-purified. HDV ribozyme sequences (blue) were transcribed in series with reselected type 5 ribozymes and cleave themselves off during transcription (Schürer et al., 2002) to yield precise 3’ ends with 2’, 3’-cyclic phosphates; the presence of this group did not affect type 5 activity. Sequences corresponding to the 5’ ‘cap+’ or ‘cap−’ regions from the selection, presenting a target for type 1 interaction, are coloured light green, with alternative arbitrary inert 5’ hairpin-forming sequences in dark green. Single-stranded sequences capable of hybridization with sites at the 3’ (or 5’, type 5cis) ends of certain templates to endow flexible ribozyme-template duplex tethering (Wochner et al., 2011; Attwater et al., 2010) are coloured yellow. Accessory domains 3’ of the catalytic core are in bold. [file elife-35255-supp1.docx]

**Supplementary file 1. Ribozyme sequences.**

All sequences are written in a 5’-to-3’ direction, generated by GMP transcription of the corresponding PCR-generated dsDNA of the sequence downstream of 5T7 sequence duplex. All transcripts were PAGE-purified.

HDV ribozyme sequences (blue) were transcribed in series with reselected type 5 ribozymes and cleave themselves off during transcription ([**Schurer et al., 2002**](#_ENREF_36)) to yield precise 3’ ends with 2’, 3’-cyclic phosphates; this presence of group did not affect type 5 activity.

Sequences corresponding to the 5’ ‘cap+’ or ‘cap−’ regions from the selection, presenting a target for type 1 interaction, are coloured light green, with alternative arbitrary inert 5’ hairpin-forming sequences in dark green. Single stranded sequences capable of hybridization with sites at the 3’ (or 5’, type 5^cis^) ends of certain templates to endow flexible ribozyme-template duplex tethering ([**Wochner et al., 2011**](#_ENREF_51)**;** [**Attwater et al., 2010**](#_ENREF_3)) are coloured yellow. Accessory domains 3’ of the catalytic core are in bold.

| Ribozyme | Sequence (5’-3’) |
| --- | --- |
| Z RPR  Z RPR^cap+^  Zcore  Zcore^cap+^ | ^p^GGAAGAAAUUCCGGACAACCAAAAAGACAAAUCUGCCCUCAGAGCUUGAGAACAUCUUCGGAUGCAGAGGAGGCAGCCUUCGGUGGCGCGAUAGCGCCAACGUUCUCAAC**AGACACCCAAUACUCCCGCUUCGGCGGGUGGGGAUAACACCUGACGAAAAGGCGAUGUUAGACACGCCCAGGUCAUAAUCCCC** |
|  | ^p^GGAUCUUCUCGAUCUGGACAACCAAAAAGACAAAUCUGCCCUCAGAGCUUGAGAACAUCUUCGGAUGCAGAGGAGGCAGCCUUCGGUGGCGCGAUAGCGCCAACGUUCUCAAC**AGACACCCAAUACUCCCGCUUCGGCGGGUGGGGAUAACACCUGACGAAAAGGCGAUGUUAGACACGCCCAGGUCAUAAUCCCC** |
|  | ^p^GGAAGAAAUUCCGGACAACCAAAAAGACAAAUCUGCCCUCAGAGCUUGAGAACAUCUUCGGAUGCAGAGGAGGCAGCCUUCGGUGGCGCGAUAGCGCCAACGUUCUCAAC |
|  | ^p^GGAUCUUCUCGAUCUGGACAACCAAAAAGACAAAUCUGCCCUCAGAGCUUGAGAACAUCUUCGGAUGCAGAGGAGGCAGCCUUCGGUGGCGCGAUAGCGCCAACGUUCUCAAC |
| Zcore selection construct (Round 1, GTP transcribed) | ^ppp^GGUCCGAAAGGACCCGCCGGUUGGCAGAACAAACAAACAGGUUGUCCAGAUCUUCUUGAUCUGGACAACCAAAAAGACAAAUCUGCCCUCAGAGCUUGAGAACAUCUUCGGAUGCAGAGGAGGCAGCCUUCGGUGGCGCGAUAGCGCCAACGUUCUCAAC**NNNNNNNNNNNNNNNNNNNNNNNNNNNNNNUGAAGAGCCUUGGUUUUUUG** |
| Type 0  0core  0core^cap+^ | ^p^GGAAGAAAUUCCGGACAGCGAAAAAGACAAAUCUGCCCUCAGAGCUUGAGAACAUCUUCGGAUGCAGAGGAGGCAGCCUUUCGGUGGCGCGAUAGCGCCAACGUCUCAAC**AAUAAAUUUCCUAGGUUCGUCACCACGUGUGAAGAGCCUUGGUUUUUUG** |
|  | ^p^GGAAGAAAUUCCGGACAGCGAAAAAGACAAAUCUGCCCUCAGAGCUUGAGAACAUCUUCGGAUGCAGAGGAGGCAGCCUUUCGGUGGCGCGAUAGCGCCAACGUCUCAAC**AA** |
|  | ^p^GGAUCUUCUCGAUCUGGACAGCGAAAAAGACAAAUCUGCCCUCAGAGCUUGAGAACAUCUUCGGAUGCAGAGGAGGCAGCCUUUCGGUGGCGCGAUAGCGCCAACGUCUCAAC**AA** |
| Type 1  1 | ^p^GGAUCUUCAUGAUCUGGAUAACCAAAAAGACCAAUCUGCCCUCAGAGCUCGAGAACAUCUUCGGAUGCAGAGGAGGCAGGCUUCGGUGGCGCGAUAGCGCCAACGUCCUCAAC**CUCCAAUGCAUCCCACCACAUGAUGAUGCCUGAAGAGCCUUGGUUUUUUG** |
|  | ^p^GACCAAUCUGCCCUCAGAGCUCGAGAACAUCUUCGGAUGCAGAGGAGGCAGGCUUCGGUGGCGCGAUAGCGCCAACGUCCUCAAC**CUCCAAUGCAUCCCACCACAUGAUGAUGCCUGAAGAGCCUUGGUUUUUUG** |
| Type 2 | ^p^GGAUCUUCUCGAUCUGGAUAACCAAAAAGACAAAUCUGUCCUCAGAGCUUGAGAGCAUCUUCGGAUGCAGAGGAGACAGCCUUCGGUGGCGCGAUAGCGCCAACGUUCUCAAC**UGGGCCAGUUAGUAAUUACACUGGACAA** |
| Type 3 | ^p^GGAUCUUCUCGAUCUGGAUAACCAAAAAGACAAACCUGAACUCAGAGCUUGAGAACAUCUUCGGAUGCAGAGGAGUCAGCCUUCGGUGGCGCGAUAGCGCCAACGUUCUCAAC**UAGUCGUGCUGUUAUAUUAUGCACGAA** |
| Type 4 | ^p^GGGUCUUCUCGACCUGGACGACCAAAAAGACAAACCUGUCCUCAGAGCUUGAGAGCAUCUUCGGAUGCAGAGGAGACAGCCUUCGGUGGCGCGAUAGCGCCAACGUUCUCAAC**CCCGAUUUCCCCCGGACU** |
| Type 5  Type 5^cap-^  Type 5^trans^  Type 5^cis^  Type 5^s^  Type 5^s^  reselection construct  (GTP transcribed)  t5  t5^a^  t5^b^  αβγδ | ^p^GGAUCUUCUCGAUCUGGACAACCAAAAAGACAAAUCUGCCAUCAAAGCUUGAGAGCAUCUUCGGAUGCAGAGGCGGCAGCCUUCGGUGGCGCGAUAGCGCCAACGUUCUCAAC**CAUGACAUGCAAAACGCGUGCUUCGUUGAAUGAAGAGCCUUGGUUUUUUG** |
|  | ^p^GGAUCUUCAUGAUCUGGACAACCAAAAAGACAAAUCUGCCAUCAAAGCUUGAGAGCAUCUUCGGAUGCAGAGGCGGCAGCCUUCGGUGGCGCGAUAGCGCCAACGUUCUCAAC**CAUGACAUGCAAAACGCGUGCUUCGUUGAAUGAAGAGCCUUGGUUUUUUG** |
|  | ^p^GGUUGCUAGAUCUUCUCGAUCUGGACAACCAAAAAGACAAAUCUGCCAUCAAAGCUUGAGAGCAUCUUCGGAUGCAGAGGCGGCAGCCUUCGGUGGCGCGAUAGCGCCAACGUUCUCAAC**CAUGACAUGCAAAACGCGUGCUUCGUUGAAUGAAGAGCCUUGGUUUUUUG** |
|  | ^p^GUCAUUGAAACAAACAAACAAGGUUGCUAGAUCUUCUCGAUCUGGACAACCAAAAAGACAAAUCUGCCAUCAAAGCUUGAGAGCAUCUUCGGAUGCAGAGGCGGCAGCCUUCGGUGGCGCGAUAGCGCCAACGUUCUCAAC**CAUGACAUGCAAAACGCGUGCUUCGUUGAAUGAAGAGCCUUGGUUUUUUG** |
|  | ^p^GGAUCUUCUCGAUCUAACAAAAAAGACAAAUCUGCCAUCAAAGCUUGAGAGCAUCUUCGGAUGCAGAGGCGGCAGCCUUCGGUGGCGCGAUAGCGCCAACGUUCUCAAC**CAUGACAUGCAAAACGCGUGCUUCGUUGAAUGAAGAGCCUUGGUUUUUUG** |
|  | ^ppp^GAAGGUCCGAAAGGACCUUCUUCUUCUUCUUCUUCUUCUUCCGGUUGGCAGAACAAACAAACAAACAAACAAACAAACAAACAGGAUCUUCUCGAUCUAACAAAAAAGACAAAUCUGCCAUCAAAGCUUGAGAGCAUCUUCGGAUGCAGAGGCGGCAGCCUUCGGUGGCGCGAUAGCGCCAACGUUCUCAAC**CAUGACAUGCAAAACGCGUGCUUCGUUGAA(1/3 N_13_, 1/3 N_20_, 1/3 N_28_)**GGGUCGGCAUGGCAUCUCCACCUCCUCGCGGUCCGACCUGGGCUACUUCGGUAGGCUAAGGGAGAAG |
|  | ^p^GGAUCUUCUCGAUCUAACAAAAAAGACAAAUCUGCCAUCAAAGCUUGAGAGCAUCUUCGGAUGCAGAGGCGGCAGCCUUCGGUGGCGCGAUAGCGCCAACGUUCUCAAC**CAUGACAUGCAAAACGCGUGCUUCGUUGAAUGGAGUUUUUCAUG**GGGUCGGCAUGGCAUCUCCACCUCCUCGCGGUCCGACCUGGGCUACUUCGGUAGGCUAAGGGAGAAG |
|  | ^p^GGAUCUUCUCGAUCUAACAAAAAAGACAAAUCUGCCAUCAAAGCUUGAGAGCAUCUUCGGAUGCAGAGGCGGCAGCCUUCGGUGGCGCGAUAGCGCCAACGUUCUCAAC**CAUGACAUGCAAAACGCGUGCUUCGUUGAAUGAAGUUUUUCAUG**GGGUCGGCAUGGCAUCUCCACCUCCUCGCGGUCCGACCUGGGCUACUUCGGUAGGCUAAGGGAGAAG |
|  | ^p^GGAUCUUCUCGAUCUGACAAAAAAGACAAAUCUGCCAUCAAAGCUUGAGAGCAUCUUCGGAUGCAGAGGCGGCAGCCUUCGGUGGCGCGAUAGCGCCAACGUUCUCAAC**CAUGACAUGCAAAACGCGUGCUUCGUUGAAUGGAGUUUUUCAUG**GGGUCGGCAUGGCAUCUCCACCUCCUCGCGGUCCGACCUGGGCUACUUCGGUAGGCUAAGGGAGAAG |
|  | ^p^GGAUCUUCUCGAUCUAACAAAAAAGACAAAUCUGCCAUCAAAGCUUGAGAGCAUCUUCGGAUGCAGAGGCGGCAGCCUUCGGUGGCGCGAUAGCGCCAACGUUCUCAAC**CAUGACAU** |
| Type 6  Type 6^s^ | ^p^GGAUCUUCUCGAUCCGGAUAACAAAAAGACAAAUCUGUCCUCAGAGCUUGAGAACAUCUUCGGAUGCAGAGGAGACAGCCUUCGGUGGCGCGAUAGCGCCAACGUUUUCAAC**ACCUGUAUCACAGGAAUUGAACCUCGAGGUGAAGAGCCUUGGUUU** |
|  | ^p^GGAUCUUCUCGAUCCAACAAAAAAGACAAAUCUGUCCUCAGAGCUUGAGAACAUCUUCGGAUGCAGAGGAGACAGCCUUCGGUGGCGCGAUAGCGCCAACGUUUUCAAC**ACCUGUAUCACAGGAAUUGAACCUCGAGGUGAAGAGCCUUGGUUU** |
