# Supplementary material for: Ribozyme-catalysed RNA synthesis using triplet building blocks
Source: eLife. 2018 May 15;7:e35255. doi: 10.7554/eLife.35255 (PMC6003772; doi:10.7554/eLife.35255)
Supplement: Supplementary file 2. — Each target was transcribed from oligonucleotides 5T7 (Supplementary file 3) and 5’-(var)-TATAGTGAGTCGTATTAATTTCGCGGGCGAGATCGATC-3’, where the (var) overhang encodes (is the DNA reverse complement of) the sequence indicated below. Guide yields: >15 nmol = ***, 10–15 nmol = **, 5–10 nmol = *, <5 nmol = ~. †: These transcriptions yield one main product suitable for use as markers to identify comigrating triplets with a similar G/AU/C content. [file elife-35255-supp2.docx]

**Supplementary file 2. Transcription of triplets.**

Each target was transcribed from oligonucleotides 5T7 (Supplementary file 3) and 5’‑(var)‑TATAGTGAGTCGTATTAATTTCGCGGGCGAGATCGATC-3’, where the (var) overhang encodes (is the DNA reverse complement of) the sequence indicated below. Guide yields: >15 nmol = ***, 10-15 nmol = **, 5-10 nmol = *, <5 nmol = ~. †: These transcriptions yield one main product suitable for use as markers to identify comigrating triplets with a similar G/AU/C content.

| Transcription target | DNA oligonucleotide 5’ overhang encodes | Product  A_260_/A_280_ | Product  yield |
| --- | --- | --- | --- |
| ^ppp^GGG | GG | 1.55 | * |
| ^ppp^GGA | GGA | 1.98 | ** |
| ^ppp^GGC | GGC | 1.44 | ** |
| ^ppp^GGU† | GGU | 1.79 | *** |
| ^ppp^GAG | CGAG | 2.08 | * |
| ^ppp^GAA | GAA | 2.72 | *** |
| ^ppp^GAC | GAC | 1.91 | * |
| ^ppp^GAU† | GAU | 2.46 | *** |
| ^ppp^GCG | GCG | 1.50 | * |
| ^ppp^GCA† | GCA | 1.98 | *** |
| ^ppp^GCC | GCC | 1.34 | ** |
| ^ppp^GCU | GCU | 1.70 | *** |
| ^ppp^GUG | GUG | 1.77 | ** |
| ^ppp^GUA | GUA | 2.45 | *** |
| ^ppp^GUC | GUC | 1.63 | ** |
| ^ppp^GUU | GUU | 2.07 | *** |
| ^ppp^AGG | CAGG | 2.10 | ** |
| ^ppp^AGA† | CAGA | 2.95 | *** |
| ^ppp^AGC | AGC | 1.99 | * |
| ^ppp^AGU | AGU | 2.51 | * |
| ^ppp^AAG | UAAG | 2.81 | *** |
| ^ppp^AAA | CAA | 4.06 | * |
| ^ppp^AAC | UAAC | 2.55 | *** |
| ^ppp^AAU | CAAU | 3.57 | ** |
| ^ppp^ACG† | ACG | 1.96 | *** |
| ^ppp^ACA | ACA | 2.85 | *** |
| ^ppp^ACC | ACC | 1.80 | * |
| ^ppp^ACU† | ACU | 2.39 | *** |
| ^ppp^AUG | AUG | 2.52 | ** |
| ^ppp^AUA | AUA | 3.92 | ** |
| ^ppp^AUC | AUC | 2.36 | ** |
| ^ppp^AUU | AUU | 3.25 | *** |
| ^ppp^CGG† | UCGG | 1.43 | ** |
| ^ppp^CGA | UCGA | 1.96 | *** |
| ^ppp^CGC | UCGC | 1.27 | ** |
| ^ppp^CGU | ACGU | 1.63 | ** |
| ^ppp^CAG | UCAG | 1.86 | ** |
| ^ppp^CAA | UCAA | 2.49 | *** |
| ^ppp^CAC | UCAC | 1.64 | ** |
| ^ppp^CAU | GCAU | 2.32 | ** |
| ^ppp^CCG | CCG | 1.30 | *** |
| ^ppp^CCA | CCA | 1.82 | ** |
| ^ppp^CCC | CC | 1.12 | * |
| ^ppp^CCU | CCU | 1.44 | ** |
| ^ppp^CUG | ACUG | 1.57 | ** |
| ^ppp^CUA† | CUA | 2.29 | ** |
| ^ppp^CUC | ACUC | 1.39 | * |
| ^ppp^CUU | CUU | 2.06 | ** |
| ^ppp^UGG | CUGG | 1.93 | *** |
| ^ppp^UGA† | CUGA | 2.58 | *** |
| ^ppp^UGC | AUGC | 1.66 | ** |
| ^ppp^UGU† | CUGU | 2.13 | *** |
| ^ppp^UAG | CUAG | 2.49 | * |
| ^ppp^UAA | CUAA | 3.75 | *** |
| ^ppp^UAC | UUAC | 2.33 | ** |
| ^ppp^UAU | CUAU | 3.27 | ** |
| ^ppp^UCG | UCG | 1.67 | * |
| ^ppp^UCA | UCA | 2.44 | ** |
| ^ppp^UCC | AUCC | 1.38 | ** |
| ^ppp^UCU | AUCU | 1.95 | ** |
| ^ppp^UUG | CUUG | 2.14 | *** |
| ^ppp^UUA | UUA | 3.42 | * |
| ^ppp^UUC | AUUC | 2.02 | * |
| ^ppp^UUU | UU | 2.82 | * |
| ^ppp^GG | GG | 1.54 | * |
| ^ppp^GA | GA | 2.50 | ** |
| ^ppp^GC | UGC | 1.42 | *** |
| ^ppp^GU | GU | 1.86 | ** |
| ^ppp^AG | AG | 2.57 | ** |
| ^ppp^AA | CAA | 4.30 | * |
| ^ppp^AC | AC | 2.26 | *** |
| ^ppp^AU | AU | 3.83 | *** |
| ^ppp^CG | UCG | 1.34 | ** |
| ^ppp^CA | UCA | 2.29 | * |
| ^ppp^CC | UCC | 1.07 | * |
| ^ppp^CU | CU | 1.59 | * |
| ^ppp^UG | CUG | 1.93 | *** |
| ^ppp^UA | CUA | 3.82 | *** |
| ^ppp^UC | UUC | 1.73 | *** |
| ^ppp^UU | CUU | 2.78 | * |
| ^ppp^GCGAAGCGUGU | GCGAAGCGUGU | N.D. | ~ |
| ^ppp^GGUCC | GGUCC | 1.53 | ** |
| ^ppp^UGACAU | CUGACAU | 2.34 | ~ |
| ^ppp^UGAAUG | CUGAAUG | 2.37 | ~ |
| ^ppp^UUCAUG | UUCAUG | 2.36 | ~ |
| ^ppp^GACAAA | GACAAA | 2.51 | * |
| ^ppp^GCAUCC | GCAUCC | 1.68 | ~ |
| ^ppp^GCCAUC | GCCAUC | 1.76 | * |
| ^ppp^CAUCUU | GCAUCUU | 1.89 | ~ |
| ^ppp^GCAAAA | GCAAAA | 2.41 | *** |
| ^ppp^GCGAUAG | GCGAUAG | 2.07 | * |
| ^ppp^UUUUUCAUG | Chemical synthesis (Chemgenes) | | |
| ^HO^CUG | Chemical synthesis (Chemgenes) | | |
| ^HO^GCG | Chemical synthesis (Chemgenes) | | |
