# Supplementary material for: Ribozyme-catalysed RNA synthesis using triplet building blocks
Source: eLife. 2018 May 15;7:e35255. doi: 10.7554/eLife.35255 (PMC6003772; doi:10.7554/eLife.35255)
Supplement: Supplementary file 3. — All sequences are written in a 5’-to-3’ direction. DNA sequences are coloured grey. RNA sequences are coloured black, with the exception of hammerhead ribozyme (brown) and HDV ribozyme (blue) sequences transcribed in series that cleave themselves off during transcription (Schürer et al., 2002) to yield precise 5’ and 3’ ends respectively. All RNAs were denaturing PAGE-purified, and DNAs were not, unless otherwise noted (‘(non)-GP’). Competing oligonucleotides, listed beneath templates, were purified using RNeasy columns (Qiagen). Primer and oligonucleotide binding sites on templates are underlined. [file elife-35255-supp3.docx]

**Supplementary file 3. Oligonucleotide sequences.**

All sequences are written in a 5’-to-3’ direction. DNA sequences are coloured grey. RNA sequences are coloured black, with the exception of hammerhead ribozyme (brown) and HDV ribozyme (blue) sequences transcribed in series that cleave themselves off during transcription ([**Schurer et al., 2002**](#_ENREF_36)) to yield precise 5’ and 3’ ends respectively. All RNAs were denaturing PAGE-purified, and DNAs were not, unless otherwise noted (‘(non)-GP’). Competing oligonucleotides, listed by templates, were purified using RNeasy columns (Qiagen). Primer and oligonucleotide binding sites on templates are underlined.

| Application | Oligonucleotide | Sequence (5’-3’)  & Origin |
| --- | --- | --- |
| Fill-in | 5T7 | GATCGATCTCGCCCGCGAAATTAATACGACTCACTATA  Sigma |
|  | HDVrt | CTTCTCCCTTAGCCTACCGAAGTAGCCCAGGTCGGACCGCGAGGAGGTGGAGATGCCATGCCGACCC  Sigma, GP |
| General primers for RNA synthesis | A9 | FITC-CUGCCAACC  IDT |
|  | A10 | FITC-CUGCCAACCG  IDT |
|  | A11 | FITC-CUGCCAACCGU  IDT |
|  | Q10 | FITC-UGCGAAGCGU  IDT |
| Primers for plus strand synthesis | Fα9 | FITC-GGAUCUUCU  IDT |
|  | Bioα9 | Biotin-GGAUCUUCU  IDT |
|  | Bioα14 | Biotin-GGAUCUUCUCGAUC  IDT |
|  | Fβ6 | FITC-GCCAUC  IDT |
|  | Fγ7 | FITC-GGAUGCA  IDT |
|  | Bioγ7 | Biotin-GGAUGCA  IDT |
|  | Bioγ11 | Biotin-GGAUGCAGAGG  IDT |
|  | Fδ7 | FITC-GCGAUAG  IDT |
|  | Fε6 | FITC-GCAAAA  IDT |
|  | Fε9 | FITC-GCAAAACGC  IDT |
| Primers for minus strand synthesis | Cyα10ba | Cy5-AGAUUUGUCU  IDT |
|  | Fβ9ba | FITC-AAGAUGCUC  IDT |
|  | Cyγ10ba | Cy5-GCCACCGAAG  IDT |
|  | Fδ7ba | FITC-AUGUCAU  IDT |
|  | Fε9ba | FITC-CAUGAAAAA  IDT |
| General templates for RNA synthesis | HTI  HTI Competing oligonucleotide | CAAUGAAUCCACGCUUCGCACGGUUGGCAGAACAGGUUGUCC  Dharmacon |
|  |  | CUGCCAACCGUGCGAAGCGUGGAUUCAUUG  Dharmacon, non-GP |
|  | I-8 | ^p^GUCAAUGACACGCUUCGCACACGCUUCGCACACGCUUCGCACACGCUUCGCACACGCUUCGCACACGCUUCGCACACGCUUCGCACACGCUUCGCACGGUUGGCAGAAAAAAAAAA  GMP transcribed from fill-in of  TTTTTTTTTTCTGCCAACCGTGCGAAGCGTGTGCGAAGCGTGTGCGAAGCGTGTGCGAAGCGTGTGCGAAGCGTGTGCGAAGCGTGTGCGAAGCGTGTGCGAAGCGTGTCATTGACTATAGTGAGTCGTATTAATTTC (IDT)  Competing oligonucleotide:  ^ppp^GGCUGCCAACCGUGCGAAGCGUGUGCGAAGCGUGUGCGAAGCGUGUGCGAAGCGUGUGCGAAGCGUGUGCGAAGCGUGUGCGAAGCGUGUGCGAAGCGUGUGCGAAGCGUGUGCGAAGCGUGUGCGAAGCGUGUCAUUGAC  Transcribed from fill-in of  GTCAATGACACGCTTCGCACACGCTTCGCACACGCTTCGCACACGCTTCGCACACGCTTCGCACACGCTTCGCACACGCTTCGCACACGCTTCGCACACGCTTCGCACACGCTTCGCACACGCTTCGCACGGTTGGCAGCCTATAGTGAGTCGTATTAATTTC  (IDT) |
|  | CCCMisAUG  CCCMisGAU  CCCMisUGA | ^p^GGGCAUGGGCGGUUGGCAGAACAAACAAACAAACAGGUUGUCC  GMP transcribed from fill-in of  GGACAACCTGTTTGTTTGTTTGTTCTGCCAACCGCCCATGCCCTATAGTGAGTCGTATTAATTTCGCGGGCGAGATCGATC  (Sigma)  Competing oligonucleotide:  ^ppp^GGAACUGCCAACCGCCCAUGCCC  Transcribed from fill-in of  GGGCATGGGCGGTTGGCAGTTCCTATAGTGAGTCGTATTAATTTCGCGGGCGAGATCGATC  (Sigma) |
|  |  | ^p^GGGAUCGGGCGGUUGGCAGAACAAACAAACAAACAGGUUGUCC  GMP transcribed from fill-in of  GGACAACCTGTTTGTTTGTTTGTTCTGCCAACCGCCCGATCCCTATAGTGAGTCGTATTAATTTCGCGGGCGAGATCGATC  (Sigma)  Competing oligonucleotide:  ^ppp^GGAACUGCCAACCGCCCGAUCCC  Transcribed from fill-in of  GGGATCGGGCGGTTGGCAGTTCCTATAGTGAGTCGTATTAATTTCGCGGGCGAGATCGATC  (Sigma) |
|  |  | ^p^GGGUCAGGGCGGUUGGCAGAACAAACAAACAAACAGGUUGUCC  GMP transcribed from fill-in of  GGACAACCTGTTTGTTTGTTTGTTCTGCCAACCGCCCTGACCCTATAGTGAGTCGTATTAATTTCGCGGGCGAGATCGATC  (Sigma)  Competing oligonucleotide:  ^ppp^GGAACUGCCAACCGCCCUGACCC  Transcribed from fill-in of  GGGTCAGGGCGGTTGGCAGTTCCTATAGTGAGTCGTATTAATTTCGCGGGCGAGATCGATC  (Sigma) |
|  | TγAGU  TγGAU | CAGACUUGCAUCC  IDT |
|  |  | CAGAUCUGCAUCC  IDT |
| 3’ to 5’ RNA synthesis | ^ppp^ba | ^ppp^GGUCCAACGUC‑FITC  Ligated by NEB T4 RNA Ligase 2 (5 µM baF, 20 µM ^ppp^GGUCC, 10 µM baSplint, 4˚C 15 h) |
|  | baF | ^p^AACGUG‑FITC  IDT  5’ phosphorylated using NEB polynucleotide kinase |
|  | baSplint | GACGTTGGACC  Sigma |
|  | T8GAA | ^p^GACGUUGGACCUUCUUCUUCUUCUUCUUCUUCUUCCGGUUGGCAGAACAAACAAACAGGUUGUCC  GMP transcribed from fill-in of  GGACAACCTGTTTGTTTGTTCTGCCAACCGGAAGAAGAAGAAGAAGAAGAAGAAGGTCCAACGTCTATAGTGAGTCGTATTAATTTC  (Sigma)  Competing oligonucleotide:  ^ppp^GGACAACCUGUUUGUUUGUUCUGCCAACCGGAAGAAGAAGAAGAAGAAGAAGAAGGUCCAACGUC  Transcribed from fill-in of  GACGTTGGACCTTCTTCTTCTTCTTCTTCTTCTTCCGGTTGGCAGAACAAACAAACAGGTTGTCCTATAGTGAGTCGTATTAATTTC  (Sigma) |
| Structured templates | SR1  SR2  SR3  SR4  SR series competing oligonucleotide | ^p^GCGUAUCGGUUGGCAGAACAAACAAACAAACAAACAGGUUGUCC  GMP transcribed from fill-in of  GGACAACCTGTTTGTTTGTTTGTTTGTTCTGCCAACCGATACGCTATAGTGAGTCGTATTAATTTCGCGGGCGAGATCGATC  (Sigma) |
|  |  | ^p^GCGUAUGCGUAUCGGUUGGCAGAACAAACAAACAAACAAACAGGUUGUCC  GMP transcribed from fill-in of  GGACAACCTGTTTGTTTGTTTGTTTGTTCTGCCAACCGATACGCATACGCTATAGTGAGTCGTATTAATTTCGCGGGCGAGATCGATC  (Sigma) |
|  |  | ^p^GCGUAUGCGUAUGCGUAUCGGUUGGCAGAACAAACAAACAAACAAACAGGUUGUCC  GMP transcribed from fill-in of  GGACAACCTGTTTGTTTGTTTGTTTGTTCTGCCAACCGATACGCATACGCATACGCTATAGTGAGTCGTATTAATTTCGCGGGCGAGATCGATC  (Sigma) |
|  |  | ^p^GCGUAUGCGUAUGCGUAUGCGUAUCGGUUGGCAGAACAAACAAACAAACAAACAGGUUGUCC  GMP transcribed from fill-in of  GGACAACCTGTTTGTTTGTTTGTTTGTTCTGCCAACCGATACGCATACGCATACGCATACGCTATAGTGAGTCGTATTAATTTCGCGGGCGAGATCGATC  (Sigma) |
|  |  | ^ppp^GGAGUUCUGCCAACCGAUACGCAUACGCAUACGCAUACGC  Transcribed from fill-in of  GCGTATGCGTATGCGTATGCGTATCGGTTGGCAGAACTCCTATAGTGAGTCGTATTAATTTCGCGGGCGAGATCGATC  (Sigma) |
|  | 4S  6S  8S | ^p^GAGAAGGAGUCCGCUCCACACCGGUUGGCAGAACAAACAAACAAACA  GMP transcribed from fill-in of  TGTTTGTTTGTTTGTTCTGCCAACCGGTGTGGAGCGGACTCCTTCTCTATAGTGAGTCGTATTAATTTC  (Sigma)  Competing oligonucleotide:  ^ppp^GGUGAAGAUUUGUUCUGCCAACCGGUGUGGAGCGGACUCCUUCUC  Transcribed from fill-in of  GAGAAGGAGTCCGCTCCACACCGGTTGGCAGAACAAATCTTCACCTATAGTGAGTCGTATTAATTTC  (Sigma) |
|  |  | ^p^GAGAAGGAGUGUCCGCACUCCACACCGGUUGGCAGAACAAACAAACAAACA  GMP transcribed from fill-in of  TGTTTGTTTGTTTGTTCTGCCAACCGGTGTGGAGTGCGGACACTCCTTCTCTATAGTGAGTCGTATTAATTTC  (Sigma)  Competing oligonucleotide:  ^ppp^GGUGAAGAUUUGUUCUGCCAACCGGUGUGGAGUGCGGACACUCCUUCUC  Transcribed from fill-in of  GAGAAGGAGTGTCCGCACTCCACACCGGTTGGCAGAACAAATCTTCACCTATAGTGAGTCGTATTAATTTC  (Sigma) |
|  |  | ^p^GAGAAGGAGUGUGUCCGCACACUCCACACCGGUUGGCAGAACAAACAAACAAACA  GMP transcribed from fill-in of  TGTTTGTTTGTTTGTTCTGCCAACCGGTGTGGAGTGTGCGGACACACTCCTTCTCTATAGTGAGTCGTATTAATTTC  (Sigma)  Competing oligonucleotide:  ^ppp^GGUGAAGAUUUGUUCUGCCAACCGGUGUGGAGUGUGCGGACACACUCCUUCUC  Transcribed from fill-in of  GAGAAGGAGTGTGTCCGCACACTCCACACCGGTTGGCAGAACAAATCTTCACCTATAGTGAGTCGTATTAATTTC  (Sigma) |
| Broccoli aptamer synthesis | BBrc10 | Biotin-C6-GGGAGACGGU  IDT |
|  | FBrcb6 | FITC-GGGAGC  IDT |
|  | TBrc | ^p^GGGAGCCCACACUCUACUCGACAGAUACGAAUAUCUGGACCCGACCGUCUCCC  GMP transcribed from fill-in of  GGGAGACGGTCGGGTCCAGATATTCGTATCTGTCGAGTAGAGTGTGGGCTCCCTATAGTGAGTCGTATTAATTTC  (Sigma) |
|  | Broc+  positive control | Biotin‑C6‑GGGAGACGGUCGGGUCCAGAUAUUCGUAUCUGUCGAGUAGAGUGUGGGCUCC  Synthesised by TGK from BBrc10 on TGKBroc+ |
|  | TGKBroc+ | GGAGCCCACACTCTACTCGACAGATACGAATATCTGGACCCGACCGTCTCCC  Sigma, GP |
| Templates for plus strand synthesis  (minus strand sequences) | TαHP  BTαHP  TαL  α synthesis competing oligonucleotide | ^p^GGUCCGAAAGGACCAGAUUUGUCUUUUUUGUCAGAUCGAGAAGAUCCAACAAACAAACAAACAAACAAACAAACAAACAGGUUGUCC  GMP transcribed from fill-in of  GGACAACCTGTTTGTTTGTTTGTTTGTTTGTTTGTTTGTTGGATCTTCTCGATCTGACAAAAAAGACAAATCTGGTCCTTTCGGACCTATAGTGAGTCGTATTAATTTC  (Sigma) |
|  |  | ^p^GGUCCGAAAGGACCAGAUUUGUCUUUUUUGUCAGAUCGAGAAGAUCCAACAAACAAACAAACAAACAAACAAACAAACAGGUUGUCCC‑(PEG)_4_-Biotin  3’ biotinylation of TαHP |
|  |  | ^p^GAUGGCAGAUUUGUCUUUUUUGUCAGAUCGAGAAGAUCC  GMP transcribed from fill-in of  GGATCTTCTCGATCTGACAAAAAAGACAAATCTGCCATCTATAGTGAGTCGTATTAATTTC  (Sigma) |
|  |  | ^ppp^GGAACAAAAGGAUCUUCUCGAUCUAACGAAAAAGACAAGUCUGGUCCUUUCGGACCG  Transcribed from fill-in of  CGGTCCGAAAGGACCAGACTTGTCTTTTTCGTTAGATCGAGAAGATCCTTTTGTTCCTATAGTGAGTCGTATTAATTTC  (Sigma) |
|  | Tβ | AAGAUGCUCUCAAGCUUUGAUGGCAACAAACAAACAAACAAACA-Biotin  IDT  Competing oligonucleotide:  ^ppp^GGAAGGACAACCUGUUUGUUUGUUUGUUUGUUUGUUGCCAUCAAGGCUUGAGAGCGUCUUG  Transcribed from fill-in of  CAAGACGCTCTCAAGCCTTGATGGCAACAAACAAACAAACAAACAAACAGGTTGTCCTTCCTATAGTGAGTCGTATTAATTTC  (Sigma) |
|  | TγHP  BTγHP  TγL  γ synthesis competing oligonucleotide | ^p^GGACCGAAAGGUCCGCCACCGAAGGCUGCCGCCUCUGCAUCCAACAAACA  GMP transcribed from fill-in of  TGTTTGTTGGATGCAGAGGCGGCAGCCTTCGGTGGCGGACCTTTCGGTCCTATAGTGAGTCGTATTAATTTC  (Sigma) |
|  |  | ^p^GGACCGAAAGGUCCGCCACCGAAGGCUGCCGCCUCUGCAUCCAACAAACAC‑(PEG)_4_-Biotin  3’ biotinylation of TγHP |
|  |  | ^p^GCUAUCGCGCCACCGAAGGCUGCCGCCUCUGCAUCCAACAAACAAACAAACAAACA  GMP transcribed from fill-in of  TGTTTGTTTGTTTGTTTGTTGGATGCAGAGGCGGCAGCCTTCGGTGGCGCGATAGCTATAGTGAGTCGTATTAATTTC  (Sigma) |
|  |  | ^ppp^GGAAGGACAACCUGUUUGUUUGUUUGUUUGUUUGUUGGAUGCAGGGGCGGCGGCCUUCGGUGGCG  Transcribed from fill-in of  CGCCACCGAAGGCCGCCGCCCCTGCATCCAACAAACAAACAAACAAACAAACAGGTTGTCCTTCCTATAGTGAGTCGTATTAATTTC  (Sigma) |
|  | Mγ^-^m1 | ^ppp^GCCACCGAAGGCUGCCGCCUCUGCAUCGGGUCGGCAUGGCAUCUCCACCUCCUCGCGGUCCGACCUGGGCUACUUCGGUAGGCUAAGGGAGAAG  3’ dephosphorylated after HDV cleavage using NEB polynucleotide kinase  Transcribed from HDV fill-in of  GATCGATCTCGCCCGCGAAATTAATACGACTCACTATAGCCACCGAAGGCTGCCGCCTCTGCATCGGGTCGGCATGGCATC  (Sigma) |
|  | Tδ  HTδ  δ synthesis competing oligonucleotide | AUGUCAUGGUUGAGAACGUUGGCGCUAUCGCAACAAACAAACAAACA-Biotin  IDT |
|  |  | GUGACAUCUGAUGAGUCCGUGAGGACGAAACGAGCUAGCUCGUCAUGUCAUGGUUGAGAACGUUGGCGCUAUCGCAACAAACAAACAAACAAACAAACAAACAGGUUGUCC  Transcribed from fill-in of  GGACAACCTGTTTGTTTGTTTGTTTGTTTGTTTGTTGCGAUAGCGCCAACGTTCTCAACCATGACATGACGAGCTAGCTCGTTTCGTCCTCACGGACTCATCAGATGTCACTATAGTGAGTCGTATTAATTTC  (IDT) |
|  |  | ^ppp^GGAAGGACAACCUGUUUGUUUGUUUGUUUGUUUGUUUGUUGCGAUAGCGCCAGCGUUCUCAGCCAUGGCAUG  Transcribed from fill-in of  CATGCCATGGCTGAGAACGCTGGCGCTATCGCAACAAACAAACAAACAAACAAACAAACAGGTTGTCCTTCCTATAGTGAGTCGTATTAATTTC  (Sigma) |
|  | Tε | CAUGAAAAACUCCAUUCAACGAAGCACGCGUUUUGCAACAAACA-Biotin  IDT  Competing oligonucleotide:  ^ppp^GGCAAAACGCGUGCUUCGUUGAAUGGAGUUUUUCAUGG  Transcribed from fill-in of  CCATGAAAAACTCCATTCAACGAAGCACGCGTTTTGCCTATAGTGAGTCGTATTAATTTC  (Sigma) |
|  | δεsplint | ^p^GUGCGUUUUGCAUGUCAUG  GMP transcribed from fill-in of  CATGACATGCAAAACGCACTATAGTGAGTCGTATTAATTTC  (Sigma) |
| Plus strand segments/ templates for minus strand synthesis | αβ | ^p^GGAUCUUCUCGAUCUGACAAAAAAGACAAAUCUGCCAUCAAAGCUUGAGAGCAUCUU  GMP-transcribed downstream of 5T7 sequence from corresponding dsDNA PCR product  Competing oligonucleotide:  TαHP |
|  | Tαba | ^p^GGGAUCUUCUCGAUCUGACAAAAAAGACAAAUCU  GMP transcribed from fill-in of  AGATTTGTCTTTTTTGTCAGATCGAGAAGATCCCTATAGTGAGTCGTATTAATTTC  (Sigma)  Competing oligonucleotide:  TαHP |
|  | Fγ | FITC-AGGAUGCAGAGGCGGCAGCCUUCGGUGGC  IDT  Competing oligonucleotide:  GCCACCGAAGGCUGCCGCCUCUGCAUCCAACAAACAAACAAACAAACA-Biotin  IDT |
|  | Mγ^+^m1 | ^ppp^GAUGCAGAGGCGGCAGCCUUCGGUGGCGGGUCGGCAUGGCAUCUCCACCUCCUCGCGGUCCGACCUGGGCUACUUCGGUAGGCUAAGGGAGAAG  Not dephosphorylated after HDV cleavage  Transcribed from HDV fill-in of  GATCGATCTCGCCCGCGAAATTAATACGACTCACTATAGATGCAGAGGCGGCAGCCTTCGGTGGCGGGTCGGCATGGCATC  (Sigma) |
|  | εHP | ^p^GGUCCGAAAGGACCGCAAAACGCGUGCUUCGUUGAAUGGAGUUUUUCAUGGGGUCGGCAUGGCAUCUCCACCUCCUCGCGGUCCGACCUGGGCUACUUCGGUAGGCUAAGGGAGAAG  GMP transcribed from HDV fill-in of  GATCGATCTCGCCCGCGAAATTAATACGACTCACTATAGGUCCGAAAGGACCGCAAAACGCGTGCTTCGTTGAATGGAGTTTTTCATGGGGTCGGCATGGCATC  (Sigma)  Competing oligonucleotide:  Tε |
|  | β+ | ^ppp^GCCAUCAAAGCUUGAGAGCAUCUUGGGUCGGCAUGGCAUCUCCACCUCCUCGCGGUCCGACCUGGGCUACUUCGGUAGGCUAAGGGAGAAG  3’ dephosphorylated after HDV cleavage using NEB polynucleotide kinase  Transcribed from HDV fill-in of  GATCGATCTCGCCCGCGAAATTAATACGACTCACTATAGCCATCAAAGCTTGAGAGCATCTTGGGTCGGCATGGCATC  (Sigma)  Competing oligonucleotide:  Tβ |
|  | δ+ | ^ppp^GCGAUAGCGCCAACGUUCUCAACCAUGACAUGGGUCGGCAUGGCAUCUCCACCUCCUCGCGGUCCGACCUGGGCUACUUCGGUAGGCUAAGGGAGAAG  3’ dephosphorylated after HDV cleavage using NEB polynucleotide kinase  Transcribed from HDV fill-in of  GATCGATCTCGCCCGCGAAATTAATACGACTCACTATAGCGATAGCGCCAACGTTCTCAACCATGACATGGGTCGGCATGGCATC  (Sigma)  Competing oligonucleotide:  Tδ |
|  | ε+ | ^ppp^GCAAAACGCGUGCUUCGUUGAAUGGAGUUUUUCAUGGGGUCGGCAUGGCAUCUCCACCUCCUCGCGGUCCGACCUGGGCUACUUCGGUAGGCUAAGGGAGAAG  3’ dephosphorylated after HDV cleavage using NEB polynucleotide kinase  Transcribed from HDV fill-in of  GATCGATCTCGCCCGCGAAATTAATACGACTCACTATAGCAAAACGCGTGCTTCGTTGAATGGAGTTTTTCATGGGGTCGGCATGGCATC  (Sigma) |
|  | δε+ | ^ppp^GCGAUAGCGCCAACGUUCUCAACCAUGACAUGCAAAACGCGUGCUUCGUUGAAUGGAGUUUUUCAUGGGGUCGGCAUGGCAUCUCCACCUCCUCGCGGUCCGACCUGGGCUACUUCGGUAGGCUAAGGGAGAAG  3’ dephosphorylated after HDV cleavage using NEB polynucleotide kinase  Transcribed from HDV fill-in of  TCGATCTCGCCCGCGAAATTAATACGACTCACTATAGCGATAGCGCCAACGTTCTCAACCATGACATGCAAAACGCGTGCTTCGTTGAATGGAGTTTTTCATGGGGTCGGCATGGCATC  (Sigma) |
|  | αβ+ | Biotin‑GGAUCUUCUCGAUCUGACAAAAAAGACAAAUCUGCCAUCAAAGCUUGAGAGCAUCUU  Synthesised by TGK from Bioα14 on TGKαβ+ |
|  | TGKαβ+ | AAGATGCTCTCAAGCTTTGATGGCAGATTTGTCTTTTTTGTCAGATCGAGAAGATCC  Sigma, GP |
|  | γδε+ | Bio‑GGAUGCAGAGGCGGCAGCCUUCGGUGGCGCGAUAGCGCCAACGUUCUCAACCAUGACAUGCAAAACGCGUGCUUCGUUGAAUGGAGUUUUUCAUG  Synthesised by TGK from Bioγ11 on TGKγδε+ |
|  | TGKγδε+ | CATGAAAAACTCCATTCAACGAAGCACGCGTTTTGCATGTCATGGTTGAGAACGTTGGCGCTATCGCGCCACCGAAGGCTGCCGCCTCTGCATCC  Sigma, GP |
| Fidelity assay extensions - encoding triplets:  Top, RNA primer (IDT)  Middle, template: GMP transcript of fill-in of DNA (Sigma)  Bottom, competing oligonuc-leotide: Transcript of fill-in of DNA (Sigma) | AAA | FITC-GCCGCCAACCGUAAAGCA,  ^p^GGGUUUGGGUGCUUUACGGAACAAACAAACAGGUUGUCC  GGACAACCTGTTTGTTTGTTCCGTAAAGCACCCAAACCCTATAGTGAGTCGTATTAATTTC,  ^ppp^GGACAACCUGUUUGUUUGUUCUGUAAAGCGCCCAAACCC  GGGTTTGGGCGCTTTACAGAACAAACAAACAGGTTGTCCTATAGTGAGTCGTATTAATTTC |
|  | AGU | FITC-GCCGCCAACCGUAGUGCA,  ^p^GGGACUGGGUGCACUACGGAACAAACAAACAGGUUGUCC  GGACAACCTGTTTGTTTGTTCCGTAGTGCACCCAGTCCCTATAGTGAGTCGTATTAATTTC,  ^ppp^GGACAACCUGUUUGUUUGUUCUGUAGUGCGCCCAGUCCC  GGGACTGGGCGCACTACAGAACAAACAAACAGGTTGTCCTATAGTGAGTCGTATTAATTTC |
|  | ACC | FITC-GCCGCCAACCGUACCGCA,  ^p^GGAGGUGGGUGCGGUACGGAACAAACAAACAGGUUGUCC  GGACAACCTGTTTGTTTGTTCCGTACCGCACCCACCTCCTATAGTGAGTCGTATTAATTTC,  ^ppp^GGACAACCUGUUUGUUUGUUCCGUACCGCACCCACCCCC  GGGGGTGGGTGCGGTACGGAACAAACAAACAGGTTGTCCTATAGTGAGTCGTATTAATTTC |
|  | GAC | FITC-GCCGCCAACCGUGACGCA,  ^p^GGGGUCGGGUGCGUCACGGAACAAACAAACAGGUUGUCC  GGACAACCTGTTTGTTTGTTCCGTGACGCACCCGACCCCTATAGTGAGTCGTATTAATTTC,  ^ppp^GGACAACCUGUUUGUUUGUUCUGUGACGCGCCCGACCCC  GGGGTCGGGCGCGTCACAGAACAAACAAACAGGTTGTCCTATAGTGAGTCGTATTAATTTC |
|  | GCG | FITC-GCCGCCAACCGUGCUGCA,  ^p^GGGCGCGGGUGCAGCACGGAACAAACAAACAGGUUGUCC  GGACAACCTGTTTGTTTGTTCCGTGCTGCACCCGCGCCCTATAGTGAGTCGTATTAATTTC,  ^ppp^GGACAACCUGUUUGUUUGUUCCGUGCUGCACCCGCGCCC  GGGCGCGGGTGCAGCACGGAACAAACAAACAGGTTGTCCTATAGTGAGTCGTATTAATTTC |
|  | GUA | FITC-GCCGCCAACCGUGUAGCA,  ^p^GGGUACGGGUGCUACACGGAACAAACAAACAGGUUGUCC  GGACAACCTGTTTGTTTGTTCCGTGTAGCACCCGTACCCTATAGTGAGTCGTATTAATTTC,  ^ppp^GGACAACCUGUUUGUUUGUUCCGUGUAGCACCCGUACCC  GGGTACGGGTGCTACACGGAACAAACAAACAGGTTGTCCTATAGTGAGTCGTATTAATTTC |
|  | CGG | FITC-GCCGCCAACCGUCGGGCA,  ^p^GGGCCGGGGUGCCCGACGGAACAAACAAACAGGUUGUCC  GGACAACCTGTTTGTTTGTTCCGTCGGGCACCCCGGCCCTATAGTGAGTCGTATTAATTTC,  ^ppp^GGACAACCUGUUUGUUUGUUCCGUCGGGCACCCCGGCCC  GGGCCGGGGTGCCCGACGGAACAAACAAACAGGTTGTCCTATAGTGAGTCGTATTAATTTC |
|  | CCA | FITC-GCCGCCAACCGUCCAGCA,  ^p^GGGUGGGGGUGCUGGACGGAACAAACAAACAGGUUGUCC  GGACAACCTGTTTGTTTGTTCCGTCCAGCACCCCCACCCTATAGTGAGTCGTATTAATTTC,  ^ppp^GGACAACCUGUUUGUUUGUUCUGUCCAGCGCCCCCACCC  GGGTGGGGGCGCTGGACAGAACAAACAAACAGGTTGTCCTATAGTGAGTCGTATTAATTTC |
|  | CUU | FITC-GCCGCCAACCGUCUUGCA,  ^p^GGGAAGGGGUGCAAGACGGAACAAACAAACAGGUUGUCC  GGACAACCTGTTTGTTTGTTCCGTCTTGCACCCCTTCCCTATAGTGAGTCGTATTAATTTC,  ^ppp^GGACAACCUGUUUGUUUGUUCUGUCUUGCGCCCCUUCCC  GGGAAGGGGCGCAAGACAGAACAAACAAACAGGTTGTCCTATAGTGAGTCGTATTAATTTC |
|  | UAU | FITC-GCCGCCAACCGUUAUGCA,  ^p^GGGAUAGGGUGCAUAACGGAACAAACAAACAGGUUGUCC  GGACAACCTGTTTGTTTGTTCCGTTATGCACCCTATCCCTATAGTGAGTCGTATTAATTTC,  ^ppp^GGACAACCUGUUUGUUUGUUCCGUUAUGCACCCUAUCCC  GGGATAGGGTGCATAACGGAACAAACAAACAGGTTGTCCTATAGTGAGTCGTATTAATTTC |
|  | UGC | FITC-GCCGCCAACCGUUGCGCA,  ^p^GGGGCAGGGUGCGCAACGGAACAAACAAACAGGUUGUCC  GGACAACCTGTTTGTTTGTTCCGTTGCGCACCCTGCCCCTATAGTGAGTCGTATTAATTTC,  ^ppp^GGACAACCUGUUUGUUUGUUCUGUUGCGCGCCCUGCCCC  GGGGCAGGGCGCGCAACAGAACAAACAAACAGGTTGTCCTATAGTGAGTCGTATTAATTTC |
|  | UUG | FITC-GCCGCCAACCGUUCGGCA,  ^p^GGGCAAGGGUGCCGAACGGAACAAACAAACAGGUUGUCC  GGACAACCTGTTTGTTTGTTCCGTTCGGCACCCTTGCCCTATAGTGAGTCGTATTAATTTC,  ^ppp^GGACAACCUGUUUGUUUGUUCCGUUCGGCACCCUUGCCC  GGGCAAGGGTGCCGAACGGAACAAACAAACAGGTTGTCCTATAGTGAGTCGTATTAATTTC |
| Fidelity assay sequencing | AdeHDVLig | A^p-p^GGGTCGGCATGGCATC-C_3_ spacer  Treatment of 20 µM HDVLig with 5’ DNA adenylation kit (NEB) 65˚C 2 h, neutral phenol/chloroform extracted and precipitated in 72% ethanol. |
|  | HDVLig | ^p^GGGTCGGCATGGCATC-C_3_ spacer  IDT, GP |
|  | HDVrec | GATGCCATGCCGACCC  Sigma, GP |
|  | P3HDV | CAAGCAGAAGACGGCATACGAGATCGGTCTCGGCATTCCTGCTGAACCGCTCTTCCGATCTGATGCCATGCCGACCC  IDT |
|  | P5GGGX | AATGATACGGCGACCACCGAGATCTACACTCTTTCCCTACACGACGCTCTTCCGATCTNNNXXXXXXGGGCTGCCAACCG  Sigma  XXXXXX = unique barcode |
| Ribozyme fragment sequencing | P5Xα8 | AATGATACGGCGACCACCGAGATCTACACTCTTTCCCTACACGACGCTCTTCCGATCTNNNXXXXXXGGGGGATCTTC  Sigma  XXXXXX = unique barcode |
|  | P5Xβ6 | AATGATACGGCGACCACCGAGATCTACACTCTTTCCCTACACGACGCTCTTCCGATCTNNNXXXXXXGGGGCCATC  Sigma  XXXXXX = unique barcode |
|  | P5Xγ7 | AATGATACGGCGACCACCGAGATCTACACTCTTTCCCTACACGACGCTCTTCCGATCTNNNXXXXXXGGGGGATGCA  Sigma  XXXXXX = unique barcode |
| Selection library synthesis | 1GMPfo  1GTPfo  1ba30N | GATCGATCTCGCCCGCGAAATTAATACGACTCACTATAGGTCCGAAAGGACCCGCCGCCGGTTGGCAGAACAAACAAACAGGTTGTCCAGATCTTCTTGATCTGGACAACCAAAAAGAC  IDT, GP |
|  |  | GATCGATCTCGCCCGCGAAATTAATACGACTCACTATAGGTCCGAAAGGACCCGCCGGTTGGCAGAACAAACAAACAGGTTGTCCAGATCTTCTTGATCTGGACAACCAAAAAGAC  IDT, GP |
|  |  | CAAAAAACCAAGGCTCTTCANNNNNNNNNNNNNNNNNNNNNNNNNNNNNNGTTGAGAACGTTGGCGCTATCGCGCCACCGAAGGCTGCCTCCTCTGCATCCGAAGATGTTCTCAAGCTCTGAGGGCAGATTTGTCTTTTTGGTTGTCCAGATCAAGAAGATCTGGACAACCTGTTTGTTTGTTC  IDT, GP |
|  | AACAt5s  T5ba13N  T5ba20N  T5ba28N | GAACAAACAAACAAACAAACAAACAAACAAACAGGATCTTCTCGATCTAAC  Sigma |
|  |  | CTTCTCCCTTAGCCTACCGAAGTAGCCCAGGTCGGACCGCGAGGAGGTGGAGATGCCATGCCGACCCNNNNNNNNNNNNNTTCAACGAAGCACGCGTTTTGC  IDT, GP |
|  |  | CTTCTCCCTTAGCCTACCGAAGTAGCCCAGGTCGGACCGCGAGGAGGTGGAGATGCCATGCCGACCCNNNNNNNNNNNNNNNNNNNNTTCAACGAAGCACGCGTTTTGC  IDT, GP |
|  |  | CTTCTCCCTTAGCCTACCGAAGTAGCCCAGGTCGGACCGCGAGGAGGTGGAGATGCCATGCCGACCCNNNNNNNNNNNNNNNNNNNNNNNNNNNNTTCAACGAAGCACGCGTTTTGC  IDT, GP |
| General selection primers | BB10PA | Dual biotin-GACUCUUCGGAGUCCUGCCAACCG  IDT (non-GP for round 1)  Competing oligonucleotide:  CCGCCGGUUGGCAGGACUCCGAAGAGUC  IDT (non-GP) |
|  | BB10PQ | Dual biotin-CAUGCGAAAGCAUGUGCGAAGCGU  IDT  Competing oligonucleotide:  AGCAGCAGCACGCUUCGCACAUGCUUUCGCAUG  IDT (non-GP) |
|  | RTri | CAAAAAACCAAGGCTCTTCA  Sigma, GP |
| Selection recovery primers | RecInt  RecIntL  RecIntQ  RecIntQL | CGGTTGGCAGAACAAACAAACAGGTTG  Sigma |
|  |  | CGGTTGGCAGAACAAACAAACAAAC  Sigma |
|  |  | ACGCTTCGCAAACAAACAAACAGG  Sigma |
|  |  | ACGCTTCGCAAACAAACAAACAAAC  Sigma |
| Selection construct synthesis | Tri2GCGM | GATCGATCTCGCCCGCGAAATTAATACGACTCACTATAGGTCCGAAAGGACCCGCCGCCGGTTGGCAGAACAAACAAACAGG  Sigma, GP |
|  | Tri3GCGM | GATCGATCTCGCCCGCGAAATTAATACGACTCACTATAGGTCCGAAAGGACCCGCCGCCGCCGGTTGGCAGAACAAACAAACAGG  Sigma, GP |
|  | Tri1UCGM | GATCGATCTCGCCCGCGAAATTAATACGACTCACTATAGGTCCGAAAGGACCCGACGGTTGGCAGAACAAACAAACAGG  Sigma, GP |
|  | Tri2UCGM | GATCGATCTCGCCCGCGAAATTAATACGACTCACTATAGGTCCGAAAGGACCCGACGACGGTTGGCAGAACAAACAAACAGG  Sigma, GP |
|  | Tri3UCGM | GATCGATCTCGCCCGCGAAATTAATACGACTCACTATAGGTCCGAAAGGACCCGACGACGACGGTTGGCAGAACAAACAAACAGG  Sigma, GP |
|  | Tri2GCUQ | GATCGATCTCGCCCGCGAAATTAATACGACTCACTATAGGCACTTCGGTGCCAGCAGCACGCTTCGCAAACAAACAAACAGGTTG  Sigma, GP |
|  | Tri3GCUQ | GATCGATCTCGCCCGCGAAATTAATACGACTCACTATAGGCACTTCGGTGCCAGCAGCAGCACGCTTCGCAAACAAACAAACAGG  Sigma, GP |
|  | Tri3CUUQ | GATCGATCTCGCCCGCGAAATTAATACGACTCACTATAGGCACTTCGGTGCCAAGAAGAAGACGCTTCGCAAACAAACAAACAGGTTG  Sigma, GP |
|  | Tri3UAUM | GATCGATCTCGCCCGCGAAATTAATACGACTCACTATAGGTCCGAAAGGACCATAATAATACGGTTGGCAGAACAAACAAACAAACAGGTTG  Sigma, GP |
|  | Tri4AAUM | GATCGATCTCGCCCGCGAAATTAATACGACTCACTATAGGTCCGAAAGGACCATTATTATTATTCGGTTGGCAGAACAAACAAACAAACAGG  Sigma, GP |
|  | Tri4ACAM | GATCGATCTCGCCCGCGAAATTAATACGACTCACTATAGGTCCGAAAGGACCTGTTGTTGTTGTCGGTTGGCAGAACAAACAAACAAACAGG  Sigma, GP |
|  | Tri5CUAQ | GATCGATCTCGCCCGCGAAATTAATACGACTCACTATAGGCACTTCGGTGCCTAGTAGTAGTAGTAGACGCTTCGCAAACAAACAAACAAACAAACAGG  Sigma, GP |
|  | Tri6CCGQ | GATCGATCTCGCCCGCGAAATTAATACGACTCACTATAGGCACTTCGGTGCCCGGCGGCGGCGGCGGCGGACGCTTCGCAAACAAACAAACAAACAAACAGG  Sigma, GP |
|  | Tri8GCAM | GATCGATCTCGCCCGCGAAATTAATACGACTCACTATAGGTCCGAAAGGACCTGCTGCTGCTGCTGCTGCTGCTGCCGGTTGGCAGAACAAACAAACAAACAAACAAACAAACAAACAGG  IDT, GP |
|  | Tri8AUAM | GATCGATCTCGCCCGCGAAATTAATACGACTCACTATAGGTCCGAAAGGACCTATTATTATTATTATTATTATTATCGGTTGGCAGAACAAACAAACAAACAAACAAACAAACAAACAGG  IDT, GP |
|  | TriGAA7GAAM | GATCGATCTCGCCCGCGAAATTAATACGACTCACTATAGAAGGTCCGAAAGGACCTTCTTCTTCTTCTTCTTCTTCTTCCGGTTGGCAGAACAAACAAACAAACAAACAAACAAACAAACAGG  IDT, GP |
| Selection rescue oligonucl-eotide | TriResc1GCGM | Biotin-GACTCTTCGGAGTCCTGCCAACCGGCG  Sigma, GP |
|  | TriResc2GCGM | Biotin-GACTCTTCGGAGTCCTGCCAACCGGCGGCG  Sigma, GP |
|  | TriResc3GCGM | Biotin-GACTCTTCGGAGTCCTGCCAACCGGCGGCGGCG  Sigma, GP |
|  | TriResc1UCGM | Biotin-GACTCTTCGGAGTCCTGCCAACCGTCG  Sigma, GP |
|  | TriResc2UCGM | Biotin-GACTCTTCGGAGTCCTGCCAACCGTCGTCG  Sigma, GP |
|  | TriResc3UCGM | Biotin-GACTCTTCGGAGTCCTGCCAACCGTCGTCGTCG  Sigma, GP |
|  | TriResc2GCUQ | Biotin-CATGCGAAAGCATGTGCGAAGCGTGCTGCTGG  Sigma, GP |
|  | TriResc3GCUQ | Biotin-CATGCGAAAGCATGTGCGAAGCGTGCTGCTGCTGG  Sigma, GP |
|  | TriResc3CUUQ | Biotin-CATGCGAAAGCATGTGCGAAGCGTCTTCTTCTTG  Sigma, GP |
|  | TriResc3UAUM | Biotin-GACTCTTCGGAGTCCTGCCAACCGTATTATTA  Sigma, GP |
|  | TriResc4AAUM | Biotin-GACTCTTCGGAGTCCTGCCAACCGAATAATAATAA  Sigma, GP |
|  | TriResc4ACAM | Biotin-GACTCTTCGGAGTCCTGCCAACCGACAACAACAACA  Sigma, GP |
|  | TriResc5CUAQ | Biotin-CATGCGAAAGCATGTGCGAAGCGTCTACTACTACTACTA  Sigma, GP |
|  | TriResc6CCGQ | Biotin‑CATGCGAAAGCATGTGCGAAGCGTCCGCCGCCGCCGCCGCCGG  Sigma, GP |
|  | TriResc8GCAM | Biotin‑GACTCTTCGGAGTCCTGCCAACCGGCAGCAGCAGCAGCAGCAGCAGCA  Sigma, GP |
|  | TriResc8AUAM | Biotin‑GACTCTTCGGAGTCCTGCCAACCGATAATAATAATAATAATAATAATA  Sigma, GP |
|  | TriResc8GAAM | Biotin‑GACTCTTCGGAGTCCTGCCAACCGGAAGAAGAAGAAGAAGAAGAAGAA  Sigma, GP |
